# *In vivo *activity of terpinen-4-ol, the main bioactive component of *Melaleuca alternifolia *Cheel (tea tree) oil against azole-susceptible and -resistant human pathogenic *Candida *species

**DOI:** 10.1186/1471-2334-6-158

**Published:** 2006-11-03

**Authors:** Francesca Mondello, Flavia De Bernardis, Antonietta Girolamo, Antonio Cassone, Giuseppe Salvatore

**Affiliations:** 1 Department of Infectious, Parasitic and Immune-mediated Diseases, Istituto Superiore di Sanità, Viale Regina Elena 299, 00161 Rome, Italy; 2 Department of Environment and Primary Prevention, Istituto Superiore di Sanità, Viale Regina Elena 299, 00161 Rome, Italy

## Abstract

**Background:**

Recent investigations on the antifungal properties of essential oil of *Melaleuca alternifolia *Cheel (Tea Tree Oil, TTO) have been performed with reference to the treatment of vaginal candidiasis. However, there is a lack of *in vivo *data supporting *in vitro *results, especially regarding the antifungal properties of TTO constituents. Thus, the aim of our study was to investigate the *in vitro *and the *in vivo *anti-*Candida *activity of two critical bioactive constituents of TTO, terpinen-4-ol and 1,8-cineole.

**Methods:**

Oophorectomized, pseudoestrus rats under estrogen treatment were used for experimental vaginal infection with azole (fluconazole, itraconazole) -susceptible or -resistant strains of *C. albicans*. All these strains were preliminarily tested for *in vitro *susceptibility to TTO, terpinen-4-ol and 1,8-cineole for their antifungal properties, using a modification of the CLSI (formerly NCCLS) reference M27-A2 broth micro-dilution method.

**Results:**

*In vitro *minimal inhibitory concentrations (MIC_90_) values were 0.06% (volume/volume) for terpinen-4-ol and 4% (volume/volume) for 1,8-cineole, regardless of susceptibility or resistance of the strains to fluconazole and itraconazole. Fungicidal concentrations of terpinen-4-ol were equivalent to the candidastatic activity. In the rat vaginal infection model, terpinen-4-ol was as active as TTO in accelerating clearance from the vagina of all *Candida *strains examined.

**Conclusion:**

Our data suggest that terpinen-4-ol is a likely mediator of the *in vitro *and *in vivo *activity of TTO. This is the first *in vivo *demonstration that terpinen-4-ol could control *C. albicans *vaginal infections. The purified compound holds promise for the treatment of vaginal candidiasis, and particularly the azole-resistant forms.

## Background

Recently, essential oils and their components distilled from vegetable materials have been found to express antimicrobial, antioxidant, pharmacological and anticancer activities [1-3]. Among the essentials oils, Australian tea tree oil (TTO) is the most important, because it demonstrated a broad spectrum of biological activities. The European Pharmacopoeia [4] and the International Standard ISO 4730 [5] require TTO to be obtained by steam distillation from the foliage and terminal branchlets of *Melaleuca alternifolia *Cheel, and to have a minimum content of 30% of terpinen-4-ol and a maximum content of 15% of 1,8-cineole. Terpinen-4-ol is the major TTO component and has shown strong antimicrobial and anti-inflammatory properties [6,7], while 1,8-cineole is probably an undesirable allergen in TTO products [8]. Despite recent investigations on the biological properties of TTO, there still remains a paucity of *in vivo *data confirming and strengthening the *in vitro *results. This particularly applies to antifungal properties of TTO constituents. In a previous paper, we demonstrated the capacity of TTO to accelerate the clearance of *Candida albicans*, a prime agent of acute and recurrent forms of vulvovaginitis [9] from experimentally infected-rat vagina. Building on this promising result we have addressed the *in vitro *and the *in vivo *anti-*Candida *activity of two critical constituents of TTO, namely terpinen-4-ol (TERP) and 1,8-cineole (CIN). This has been done with standardised methods and the use of a recognised model of estrogen-induced vaginal candidiasis in rats [10].

## Methods

### *Melaleuca alternifolia *Cheel (tea tree) oil and components

Australian *Melaleuca alternifolia *(Maiden and Betch) Cheel oil, supplied by Variati (Milan, Italy), was analysed for the exact determination of single constituents and the correct conditions for storage, as shown in the Results (see Table 1). Terpinen-4-ol purchased from Fluka (Buchs, Switzerland) and 1,8-cineole from Sigma-Aldrich (St Louis, MO, USA), were used as positive markers. All components used were > 97% pure.

**Table 1:** Chemical composition of the *Melaleuca alternifolia *Cheel essential oil

Components	Kovats Index, K.I.^a^	Percentage, %
α-Thujene	931	0.94
α-Pinene	939	2.42
Sabinene	976	0.40
β-Pinene	980	0.73
Myrcene	991	0.87
α-Phellandrene	1005	0.34
α-Terpinene	1018	9.76
p-Cymene	1026	2.82
Limonene	1031	1.75
1,8-Cineole	1033	3.57
γ-Terpinene	1062	20.65
Terpinolene	1088	3.71
Terpinen-4-ol	1168	42.35
α-Terpineol	1189	3.09
Aromadendrene	1439	0.94
δ-Cadinene	1524	1.05

^a^The substances were reported in increasing order of Retention Time expressed as Kovat's Index

### Gas chromatography (GC-FID) and gas-chromatography-mass spectrometry (GC-MS)

Gas chromatography appliances used included a Perkin Elmer Auto System equipped with two fused-silica SPB columns (60 m × 0.25 mm i.d.; film thickness 0.25 μm), mounted in parallel in the same oven, with two detectors: FID and Q-Mass 910 (electron ionization 70 eV electron energy, transfer line 220°C). Carrier gas was oxygen and moisture-free helium obtained from a SUPELCO High Capacity Heated Carrier Gas Purifier (Sigma-Aldrich, Milan), provided with an OMI-2 indicating tube, at an average flow rate of 1 mL/min. The oven temperature programme was 60°C for 4 min, then 2°C/min until 180°C was reached, then increased 3°C/min until 250°C. The detector and the injector temperature was 280°C. The volume of injected essential oil or pure substance was 0.1 μL, and the split ratio was 1:50. Two distinct data systems were connected to the GC-FID or GC-MS: Turbochrom and Q-mass Analytical Workstation Software (Perkin-Elmer, Milan) with a NIST/EPA/MSDC Mass Spectral database.

### Antifungal agents

A stock solution of fluconazole (FLC) technical grade (5000 mg/L; Pfizer Inc., NY, USA) was prepared in sterile distilled water, and a stock solution of itraconazole (ITC) technical grade (1000 mg/L; Janssen Pharmaceutica, Beerse, Belgium) was prepared in polyethylene glycol 400, by heating at 75°C for 45 min.

### Yeast isolates

A total of 49 clinical isolates of *C. albicans *were used throughout this study. All were isolated from oropharyngeal swabs of HIV-seropositive subjects. Seven isolates were resistant to FLC, 13 to ITC and 6 to both drugs, as established by international breakpoint standards [11]. All clinical isolates were identified according to morphology on corn meal agar, followed by germ tube formation and assimilation-fermentation profiles in the API 20 system (bioMérieux, Marcy l'Étoile, France), as reported elsewhere [12]. Reference strains were also used for comparative purposes, as listed in Table 2. *Candida parapsilosis *ATCC 22019 and *Candida krusei *ATCC 6258 were also quality control (QC) isolates.

**Table 2:** *In vitro *antifungal activity of tea-tree oil, terpinen-4-ol, 1,8-cineole and comparators^a ^in reference strains

Organism	FLC	ITC	TTO	TERP		CIN	
					
	MIC mg/L	MIC mg/L	MIC %v/v	MIC %v/v	MFC %v/v	MIC %v/v	MFC %v/v
*C. albicans *ATCC 24433	0.25	0.06	0.25	0.06	0.125	> 4.0	> 4.0
*C. albicans *ATCC 76615	0.125	0.06	0.25	0.03	0.125	> 4.0	> 4.0
*C. albicans *ATCC 90029	0.125	0.03	0.25	0.03	0.125	4.0	4.0
*C. albicans *ATCC 10231	0.125	0.03	0.25	0.06	0.06	> 4.0	> 4.0
*C. tropicalis *ATCC750	1.0	0.125	0.06	0.03	0.125	> 4.0	> 4.0
*C.parapsilosis *ATCC 22019	2.0	0.06	0.125	0.03	0.125	> 4.0	> 4.0
*C. krusei *ATCC 6258	32.0	0.25	0.25	0.03	0.125	> 4.0	> 4.0
*C. glabrata *ATCC 90030	4.0	0.125	0.06	0.03	0.125	2.0	> 4.0
*S. cerevisiae *ATCC 9763	4.0	0.25	n.a.	0.03	0.125	4.0	> 4.0
*C.neoformans *ATCC 90112	2.0	0.03	0.03	0.015	0.06	2.0	> 4.0
*C. neoformans *ATCC 90113	4.0	0.125	n.a.	0.015	0.06	1.0	n.a.

^a^FLC = Fluconazole; ITC = Itraconazole; n.a. = not assessed

### Determination of minimum inhibitory and fungicidal concentration

Susceptibility testing of *C. albicans *and other yeasts to TTO, terpinen-4-ol, 1,8-cineole, FLC and ITC was performed according to the Clinical and Laboratory Standards Institute (CLSI, formerly NCCLS) method for broth dilution antifungal susceptibility testing of yeasts [11].

Each antifungal compound was diluted using RPMI 1640 medium with L-glutamine, without sodium bicarbonate (Sigma Chemical Co., St Louis, MO, USA) and buffered to pH 7.0 with 0.165 M 4-Morpholinepropanesulfonic acid (MOPS) buffer (Sigma). Aliquots of 50 μL of two-fold dilutions of drug solutions were dispensed in 96-well microtitre plates. The final concentration of the antifungal agents ranged from 0.0078 to 4 mg/L for ITC, 0.125 to 64 mg/L for FLC and 0.0078% to 4% v/v for TTO, terpinen-4-ol and 1,8-cineole. Tween-80 (final concentration 0.001% v/v) was included to facilitate oil solubility [13]. At this concentration, no inhibitory effect on yeast growth was shown by the detergent. The cell density of the suspensions was estimated by direct cell count using a Thoma camera, and adjusted to a cell density ranging from 0.5 × 10^3 ^to 2.5 × 10^3 ^cfu/mL (twice the final inoculum size); 50 μL was added to each well of the micro-dilution plate, followed by incubation for 48 hr at 35 C°. Minimum inhibitory (MIC) and fungicidal concentrations (MFC) were determined. The MIC was defined as the lowest concentration that produced a 50% reduction of growth, compared with growth of the drug-free control growth, and the MFC as the lowest drug concentration resulting in the death of 99.9% or more of the initial inoculum. To determine MFCs, 10 μL of broth was taken from the well without microbial growth, inoculated onto Sabouraud's dextrose agar (SDA) and incubated at 35°C. After 48 hr the cfu were counted to assess viability. Each experiment was performed in triplicate, independently. The minimum concentration of drug that inhibited 90% and 50% of the isolates tested was defined as MIC_90 _and MIC_50_. The criteria for definition of susceptibility/resistance to FLC/ITC were those established by the CLSI [11].

### Experimental vaginal infection

A rat vaginal model was used for the experimental vaginal infection, as previously described [14]. Experiments were carried out with FLC-susceptible and -resistant strains of *C. albicans*. Two independent experiments with each fungal strain were conducted and in each experiment groups of five rats were used. Oophorectomized female Wistar rats (80–100 g; Charles River Calco, Italy) were injected subcutaneously with oestradiol benzoate 0.5 mg (Estradiolo, Amsa Farmaceutici srl, Rome, Italy). Six days after the first oestradiol dose, all animals were inoculated intravaginally with 10^7 ^yeast cells of each *C. albicans *strain tested in 0.1 mL of saline. The strains used for the challenge were *C. albicans *SA-40, which was susceptible to both FLC and ITC, and AIDS-68, which was resistant to both these drugs. The inoculum was dispensed into the vaginal cavity through a syringe equipped with a multipurpose calibrated tip (Combitip; PBI, Milan, Italy). The yeast cells had been grown previously in YPD broth (yeast extract 1%, peptone 2%, dextrose 2%) at 28°C on a gyrator shaker (200 rpm), harvested by centrifugation (1500 g), washed, counted in a haemocytometer, and suspended to the required number in saline solution. The number of cells in the vaginal fluid was counted by culturing 1 μL samples (using a calibrated plastic loop, Disponoic; PBI) taken from each animal, on SDA containing chloramphenicol (50 mg/L) as previously described. The kinetics of *Candida *vaginal infection were monitored by the number of cfu/mL of vaginal lavage fluid. TTO and terpinen-4-ol were administered intravaginally (0.1 mL at 1%, 2.5% and 5%, in 0.001% Tween-80 for TTO; 0.1 mL at 1%, in 0.001% Tween-80 for terpinen-4-ol), at 1, 24 and 48 hr after intravaginal *C. albicans *challenge. Rats receiving FLC (3 doses of 100 μg intravaginally) or Tween-80 served as positive or negative controls, respectively. The infection was monitored for at least 21 days after the challenge, with vaginal fluid sampling usually being made at 1, 24 and 48 hr, then on days 5, 7, 14 and 21. The animal experimentation referred to in this paper was approved by the *ad hoc *committee of the Istituto Superiore di Sanità, Rome, Italy.

### Statistical analysis

The significance of mean cfu differences in the vaginal infection was assessed by Student's *t*-test and set at *P *< 0.05 (two tailed).

## Results

### Chemical identification and quantitative estimations

TTO composition was determined by comparing GC retention times, the Kovat's Indices (15) and GC/MS spectra with those of the co-injected reference substances. In the absence of reference substances, the structure of the components was tentatively assigned by the Officinal NIST/EPA/MSDL Spectral Library. Quantitative data were based on peak area normalisation without using a correction factor. The substances and their relative composition are shown in Table 1. The oil was a terpinen-4-ol type according to the European Pharmacopoeia [4] and the International Standard ISO 4730:1996 [5].

### Antifungal activity

Terpinen-4-ol and 1,8-cineole were compared with the mother oil for their antifungal activity *in vitro*. This activity included FLC and ITC resistant isolates, as shown in Tables 2 and 3. Terpinen-4-ol and 1,8-cineole inhibited all isolates tested, though with different MIC values (Table 3). In fact the MICs ranged from 0.015% to 0.06% for terpinen-4-ol and from 1% to > 4% v/v for 1,8-cineole. MIC_90 _values were 0.06% for terpinen-4-ol and 4 % v/v for 1,8-cineole, regardless the azole-susceptibility or resistance of the strains. Overall, the antifungal activity (MICs_90_) *in vitro *was twice higher for terpinen-4-ol (0.06% v/v) and four times lower for 1,8-cineole (4% v/v), in comparison to TTO activity (0.25% v/v) in susceptible strains of *C. albicans*, while it was (MIC_90_) three times higher for terpinen-4-ol (0.06% v/v) and three times lower for 1,8-cineole (4% v/v), in comparison to TTO activity (0.5% v/v) in resistant strains of *C. albicans*. Terpinen-4-ol was also fungicidal as determined by MFC.

**Table 3:** *In vitro *anti-*C.albicans *activity of tea tree oil, terpinen-4-ol, 1,8-cineole and comparators^a^

MIC^b^	Drug	Organism	
		
		*Candida albicans*^c ^(35)^e^	*Candida albicans*^d ^(14)^e^
MIC_50_	FLC	0.125	64.0 (7)^d^
	ITC	0.03	4.0 (13)
	TTO	0.125	0.25
	TERP	0.03	0.06
	CIN	2.0	4.0
MIC_90_	FLC	0.25	64.0
	ITC	0.03	4.0
	TTO	0.25	0.5
	TERP	0.06	0.06
	CIN	4.0	4.0
MIC range	FLC	0.125–2.0	32.0–64.0
	ITC	0.0078-0.5	4.0
	TTO	0.06-0.5	0.25-0.5
	TERP	0.015-0.06	0.03-0.06
	CIN	1.0>4.0	> 4.0

^a^Fluconazole (FLC); Itraconazole (ITC); ^b^MICs are expressed in mg/L (FLC and ITC) or in % v/v (TTO, TERP, CIN); ^c^FLC- and ITC- susceptible isolates. The MICs for the QC of *C. krusei *were 32 mg/L for FLC and 0.25 mg/L for ITC, 0.25% v/v for TTO, 0.06% v/v for TERP and ≥ 4% v/v for CIN. The MICs for the QC of *C. parapsilosis *were 2.0 mg/L for FLC and 0.06 mg/L for ITC, 0.125% v/v for TTO, 0.03 % v/v for TERP and 4% v/v for CIN; ^d^FLC, ITC or both, resistant isolates; six isolates were cross resistant; ^e^In parenthesis, the number of strains tested.

Generally, MFCs_90 _and MICs_90 _of terpinen-4-ol (0.06% v/v) coincided in azole susceptible strains. In contrast, MFCs_90 _(0.5% v/v) were three times higher for azole resistant strains of *C. albicans *(Table 4). The component 1,8-cineole was also fungicidal as determined by MFC. Table 3 also shows the data for the mother compound mixture TTO, which confirms previously published data by our group [10]. The MIC values for TTO ranged from 0.06% to 0.5% v/v. MICs_90 _were 0.25% v/v for azole-susceptible and 0.5% v/v for azole-resistant *C. albicans *strains. At the MIC value and confirming previous results [10], TTO was generally also fungicidal, as determined by MFC. The MIC and MFC coincided for each isolate. TTO was also active against FLC-, ITC-resistant strains or both, with MICs_50 _of 0.25% v/v and MICs_90 _of 0.5% v/v, for both drugs. In Table 3, MICs for FLC, ITC, TTO, terpinen-4-ol, 1,8-cineole are shown for comparative purposes.

**Table 4:** *In vitro *fungicidal activity of tea-tree oil, terpinen-4-ol, 1,8-cineole and comparators^a^

		MFC_50_					MFC_90_				
Organism	No. of isolates	FLC (mg/L)	ITC (mg/L)	TTO (%v/v)	TERP (%v/v)	CIN (%v/v)	FLC (mg/L)	ITC (mg/L)	TTO (%v/v)	TERP (%v/v)	CIN (%v/v)

*Candida albicans*^b^	35	> 64	> 4	0.25	0.03	2	> 64	> 4	0.25	0.06	2
*Candida albicans*^c^	14	> 64(7)^c^	> 4(13)	0.5	0.25	4	> 64	> 4	0.5	0.5	4

^a^Fluconazole (FLC); Itraconazole (ITC);^b ^FLC- and ITC- susceptible isolates. The MICs for the QC of *C. krusei *were 32 mg/L for FLC and 0.25 mg/L for and ITC, 0.25% v/v for TTO, 0.06% v/v for TERP and ≥ 4% v/v for CIN. The MFC was 0.125% for TERP. The MICs for the QC of *C. parapsilosis *were 2 mg/L for FLC and 0.06 mg/L for ITC, 0.125% v/v for TTO, 0.03 % v/v for TERP and 4% v/v for CIN. The MFC was 0.125 % v/v for TERP; ^c^FLC, ITC resistant isolates or both; six isolates were cross resistant.

### Experimental vaginal infection

**Figure 1:**
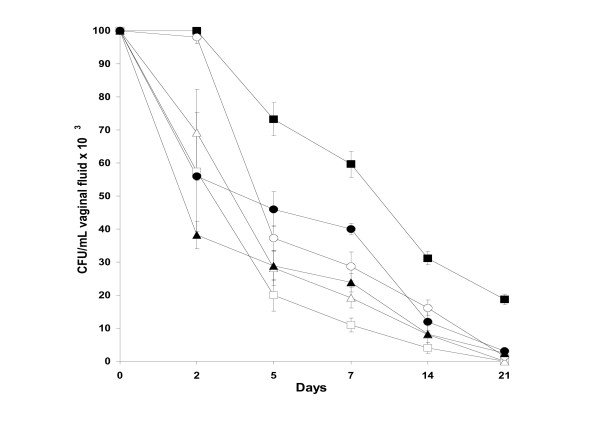
Vaginal infection outcome by a fluconazole-susceptible strain *C. albicans *(SA-40) in oophorectomized, oestradiol-treated rats inoculated intravaginally with TTO 5% v/v (open squares), 2.5% v/v (open triangles), 1% v/v (open circles), terpinen-4-ol 1% v/v (filled triangles), fluconazole 100 μg (filled circles), Tween-80 0.001% v/v (control; filled squares) at 1, 24 and 48 hr after intravaginal *C. albicans *challenge (10^7 ^cells in 0.1 mL). Each curve represents the mean (± S.E.) of cfu of five rats. Data are from one of two independent experiments with similar results. At each time point considered, starting from day 2 to day 14, there was a statistically significant difference between the cfu of rats treated with fluconazole or TTO 5% or TTO 2.5% or terpinen-4-ol and those of the untreated animals.

**Figure 2:**
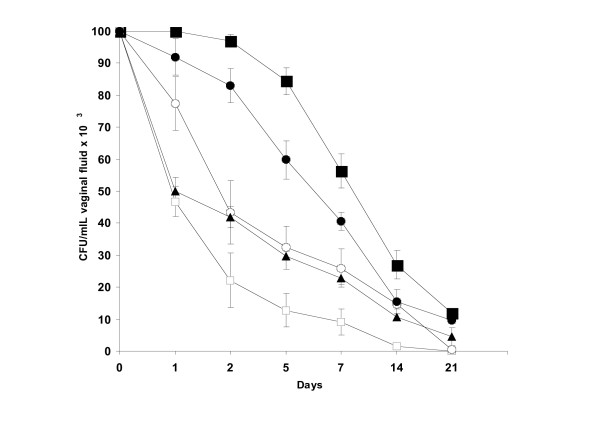
Vaginal infection outcome by fluconazole-itraconazole resistant strain *C. albicans *(AIDS-68) in oophorectomized, oestradiol-treated rats inoculated intravaginally with TTO 5% v/v (open squares), 1% v/v (open circles), terpinen-4-ol 1% v/v (filled triangles), fluconazole 100 μg (filled circles), Tween-80 0.001% v/v (control; filled squares) at 1, 24 and 48 hr after *C. albicans *challenge (10^7 ^cells in 0.1 mL). Each curve represents the mean (± S.E.) of the fungal cfu of five rats. At each time point (day1-day14) there was a statistically significant difference (P < 0.05, Student's *t *test, two tailed) between the untreated control and cfu of rats treated with TTO 5%, TTO 1% and terpinen-4-ol. At scattered day intervals, there was also a statistically significant difference (P < 0.05, Student's *t *test, two tailed) between cfu of rats treated with TTO 5% or terpinen-4-ol and TTO 1% (for example day 1).

After establishing activity *in vitro*, we examined and compared the activity of terpinen-4-ol with the mother mixture TTO *in vivo*. Cineole was excluded from this investigation because of its weak *in vitro *antifungal activity. We selected an experimental mucosal infection (oestrogen-dependent rat vaginitis) in which the animals were challenged with either a FLC-ITC-susceptible (SA-40) or a FLC-resistant (AIDS-68) *C. albicans *strain. Two experiments were performed with each strain, and these produced substantially overlapping results. Figures 1 and 2 show the details of one of the two experiments. As shown in Figure 1, which refers to results obtained with FLC-susceptible (SA-40) *C. albicans *strain, terpinen-4-ol (1% v/v) exerted a marked acceleration of clearance of the yeast, as demonstrated by a statistically significant decrease in cfu counts in the first 2 weeks after the vaginal challenge, compared with the control (terpinen-4-ol-untreated animals, only given the Tween-80 diluent). As with all dose regimens, the infection was cleared in 3 weeks, whereas the untreated control rats remained infected (approximately 2.5 × 10^4 ^*C. albicans *cfu/mL of vaginal fluid). FLC treatment, used as a positive control, showed a pattern of clearance comparable to that induced by terpinen-4-ol. No effect on the rate of fungal clearance was observed in rats treated with terpinen-4-ol diluent Tween-80. In comparative terms, the acceleration of *Candida *clearance in rats treated with 1% terpinen-4-ol solution did substantially overlap the activity of a 5% v/v solution of TTO and statistically significant compared to 1% v/v TTO. As shown in Figure 2, terpinen-4-ol (1% v/v) also caused a rapid clearance of the FLC-resistant strain from the vagina of experimentally infected rats. In this case however, TTO (5% v/v) was significantly more active than terpinen-4-ol. Also of note, the *in vitro *resistance to FLC did not cause the strain to be totally unaffected by FLC *in vivo*.

## Discussion

As stated elsewhere [10] the interest in non-conventional, non-prescription natural medicinals in the field of infection parallels an increased awareness of side effects of conventional drugs. There is also a necessity in finding new approaches in the therapy of infections in an era of emerging and remerging infections and the spread of antimicrobial drug resistance. In this line of research, we have previously shown that one of the most popular natural medicaments, namely TTO, had a significant curative effect on experimental vaginal candidiasis in rats [10] in keeping with a remarkable antifungal activity *in vitro*, also previously shown also by other authors [16]. However, no dissection of the individual constituents was made in previous studies, and we could not attribute the anticandidal *in vivo *activity to any of the numerous TTO constituents. Based on preliminary observations indicating that 1,8-cineole and terpinen-4-ol could be involved and are mostly responsible for this activity, we have in this study separated these different constituents and investigated their effects. We specifically examined whether terpinen-4-ol could mimic the activity of the whole TTO in exerting a therapeutic effect against both azole-susceptible and azole-resistant strains of *C. albicans in vivo*. Here we show that terpinen-4-ol rather than 1,8-cineole is the most likely mediator of TTO activity or, at least, a main contributor of it. Our *in vitro *data confirm that terpinen-4-ol has the highest antimicrobial activity and, in contrast, 1,8-cineole exhibited a much lower activity [13,17]. The terpinen-4-ol MIC_90 _and MFC_90 _for *C. albicans *strains determined in our study did not match those reported by other authors against the same fungus [17-20]. MIC_90 _and MFC_90 _for terpinen-4-ol were lower than those of TTO. Importantly, the MICs_90 _were always lower even for resistant *C. albicans *strains. It is of interest that the batch of terpinen-4-ol used had a fungicidal concentration (0.5% v/v) not equal to the MIC (0.06% v/v) for resistant *C. albicans *strains, thus demonstrating the rapid cytocidal activity of this main component. This was also demonstrated by the time-kill experiments, as shown by others [21]. The interpretation of susceptibility was easy because a distinct endpoint of growth inhibition was produced without trailing growth [22].

In this study, we have specifically compared the therapeutic activity of TTO and terpinen-4-ol in a well-established experimental model of rat vaginal candidiasis, in which the effect of immunotherapy by passive transfer of antibodies, or active vaccination with whole *Candida *cells or subunit antigens, has been assessed extensively [23-25]. This model has also been shown to be a valuable tool in determining and predicting the antifungal activity of various drugs, including the HIV-protease inhibitors [26]. This investigation was in part instigated by the numerous claims and anecdotal reports on the therapeutic activity of TTO against vaginal infections, including vaginal candidiasis. A potential advantage of novel therapeutics is their capacity to inhibit micro-organisms that are resistant to existing drugs; we therefore tested the *in vivo *activity of TTO and terpinen-4-ol against a strain of *C. albicans *resistant to FLC, one of the most popular and medically effective anti-*Candida *drugs. The results of our investigations demonstrate that terpinen-4-ol treatment is efficacious in substantially accelerating the experimental vaginal infection by *C. albicans *with both FLC-susceptible and -resistant isolates. In the case of the drug-susceptible organism, treatment with terpinen-4-ol was comparable to a standard treatment with FLC. In all cases, the infection was resolved (using 1% v/v terpinen-4-ol) by the third week of treatment. Importantly, terpinen-4-ol treatment was equally efficacious against a azole-susceptible as well as against a FLC-ITC resistant organism. Throughout this investigation, there was no evidence of suffering by the animals under terpinen-4-ol treatment, or any sign of allergic response to a treatment that was easily dispensed and non-chronic in nature (one intravaginal application a day, for only the first 3 days, after intravaginal challenge).

## Conclusion

Overall, our experimental data strengthen the previous contribution [10] on TTO activity *in vivo*. In particular, we have now identified for the first time terpinen-4-ol as a single active *in vivo *constituent of TTO mixture. This highlights the therapeutic anti-*Candida *potential of a purified, single component, thus avoiding the necessity of a laborious and costly quality control of a mixture of compounds. A clear need remains for pre-clinical and clinical investigations aimed at a more extensive assessment of terpinen-4-ol, including studies on the mechanisms of anticandidal activity.

## Competing interests

The author(s) declare that they have no competing interests.

## Authors' contributions

FM was primarily involved in the conceptual planning of the paper and achievement *in vitro *data. FDB was responsible for *in vivo *data. AG investigated the azole-resistant strains *in vitro*. AC contributed to the analysis and interpretation of the data. GS was responsible for the chemical analysis and general supervision of the paper.

## Pre-publication history

The pre-publication history for this paper can be accessed here:


